# Genome wide discovery of long intergenic non-coding RNAs in Diamondback moth (*Plutella xylostella*) and their expression in insecticide resistant strains

**DOI:** 10.1038/srep14642

**Published:** 2015-09-28

**Authors:** Kayvan Etebari, Michael J. Furlong, Sassan Asgari

**Affiliations:** 1 School of Biological Sciences, The University of Queensland, Brisbane QLD 4072 Australia

## Abstract

Long non-coding RNAs (lncRNAs) play important roles in genomic imprinting, cancer, differentiation and regulation of gene expression. Here, we identified 3844 long intergenic ncRNAs (lincRNA) in *Plutella xylostella*, which is a notorious pest of cruciferous plants that has developed field resistance to all classes of insecticides, including *Bacillus thuringiensis* (Bt) endotoxins. Further, we found that some of those lincRNAs may potentially serve as precursors for the production of small ncRNAs. We found 280 and 350 lincRNAs that are differentially expressed in Chlorpyrifos and Fipronil resistant larvae. A survey on *P. xylostella* midgut transcriptome data from Bt-resistant populations revealed 59 altered lincRNA in two resistant strains compared with the susceptible population. We validated the transcript levels of a number of putative lincRNAs in deltamethrin-resistant larvae that were exposed to deltamethrin, which indicated that this group of lincRNAs might be involved in the response to xenobiotics in this insect. To functionally characterize DBM lincRNAs, gene ontology (GO) enrichment of their associated protein-coding genes was extracted and showed over representation of protein, DNA and RNA binding GO terms. The data presented here will facilitate future studies to unravel the function of lincRNAs in insecticide resistance or the response to xenobiotics of eukaryotic cells.

Metazoan genomes encode a large number of regulatory and housekeeping noncoding RNAs (ncRNAs). Regulatory ncRNAs include small RNAs, such as microRNAs (miRNAs), small interfering RNAs (siRNAs), and long noncoding RNAs (lncRNAs), which are mainly involved in regulation of gene expression. RNA transcripts longer than 200 nucleotides, which do not contain an open reading frame of longer than 100 amino acids, are simply defined as lncRNA^1^,^2^. Although several lncRNAs have been identified and characterized in the last decade, genome-wide identification of this class of ncRNAs has only recently become possible with the advent of whole transcriptome sequencing technologies.

Generally, they are classified by their location relative to their neighboring protein-coding genes and include the long intergenic ncRNA (lincRNA), intronic lncRNA, antisense lncRNA and enhancer RNA^1^. However, some other nomenclature has been suggested in the light of the rising amount of deep sequencing data, which provides more information on lncRNA function and mechanism. Bonasio and Shiekhattar (2014) classified lncRNAs to promoter-associated RNA (pRNA), enhancer-associated RNA (eRNA), intervening RNA (iRNA) and gene body associated RNAs which included sense (gsRNA) and antisense overlapping non coding RNA (gaRNA)^3^.

RNA polymerase II is responsible for the transcription of lncRNA and usually some of these transcripts are precursors for small regulatory RNAs, but a large number of them have no distinguishable purpose^4^. However, understanding the biology of lncRNAs is still at an early stage and there are disagreements regarding their functionality and classification^3^. Although it has been suggested that lncRNAs are merely products of inaccuracy in transcription machinery or transcriptional noise^5^, their involvement in several biological pathways has been demonstrated^6^,^7^,^8^. Considering all possible scenarios, even if only 10% of identified lncRNAs have a biological role, more than a thousand human loci would generate functional lncRNAs^4^.

Recent findings have demonstrated that lncRNAs take part in genomic imprinting, cancer, cell differentiation, regulation of gene expression and development^9^,^10^,^11^,^12^,^13^,^14^. Databases have also been developed that facilitate exploring interactions of ncRNAs, including lncRNAs, with proteins, RNAs and viruses, but mainly in humans^15^,^16^. While studies on insect lncRNAs are limited, they suggest that lncRNAs could be involved in various functions. For example, it has been shown that *Apis mellifera* lncRNAs, which are highly expressed in ovaries, are probably involved in the fine tuning of developmental processes underlying phenotypic plasticity related to social life histories in honey bees^9^. It has also been reported that heterochromatin formation in insects is correlated with lncRNA expression^12^. Overexpression of lncRNA in certain developmental stages of *Spodoptera frugiperda* produced a variety of lncRNA associated small RNAs, such as repeat associated small interfering RNAs (rasiRNAs), which appeared to correlate with subsequent accumulation of a heterochromatic histone mark^12^. Recently, it was shown that lncRNAs also coordinate sex determination in *Drosophila*^17^.

Previous work has indicated linRNAs’ quick response to different stimuli and stress factors^8^,^18^,^19^. Up-regulation of some lncRNAs by genotoxic agents such as mitomycin C or doxorubicin has been reported in a few mammalian cell lines^18^. Some lincRNAs act as suppressors of the p53 transcriptional response and they were induced by DNA damage caused by doxorubicin^20^. Stress can also change the lincRNA expression profile in plants and in *Arabidopsis thaliana*, the expression level of a lincRNA (At5NC056820) increased by 22-fold when plants were treated with elf18 (EF-Tu), which triggers pathogen-associated molecular pattern responses^21^. The involvement of lncRNAs in pathways associated with responses to cellular stress makes them interesting candidates to investigate when organisms are exposed to insecticides and other toxicants^22^.

lncRNAs demonstrate low evolutionary sequence conservation even among closely related species. However, significant improvement in RNAseq technologies and reproducibility-based bioinformatics strategies for genome-wide screening have uncovered thousands of lncRNA sequences in the genomes of both lower living organisms, such as yeasts, as well as higher eukaryotes. For instance, more than 14,000 lncRNA genes in human^4^,^23^, 6480 in *A. thaliana*^21^, 4000 in bovine muscle^24^ and 1133 multi-exonic lncRNA transcripts from zebrafish (*Danio rerio*)^25^ have been reported in the last few years. Among these, only a few genes have been annotated as lncRNA in insects, which are mostly in model species such as *Drosophila melanogaster* and *A. mellifera.* It has been estimated that more than 5000 loci potentially encode non-coding transcripts in *D. melanogaster*, however, only seven loci (*bxd, Hsrω, pgc, roX1, rox2, sphinx* and *yar*) have been experimentally annotated as functional regulatory lncRNAs in the insect^26^. A recent study conducted by Padron *et al.* (2014) demonstrated that 43% of total midgut transcripts of *Anopheles gambiae* are lncRNAs and 32% of them showed some level of homology to other species^27^.

Diamondback moth (DBM), *Plutella xylostella* L (Lepidoptera: Plutellidae) is a notorious pest of cruciferous plants that has developed resistance to all groups of insecticides, including *Bacillus thuringiensis* endotoxins^28^. This has contributed towards *P. xylostella* becoming one of the world’s most destructive insect pests and its estimated cost to global economy has been estimated to be US$ 4–5 billion annually^28^,^29^. This emphasizes the necessity for the continued development of innovative alternative control measures and resistance management strategies.

The current study provides a glimpse of the lincRNA profile of this important agricultural insect pest. This catalog, as the first list of *P. xylostella* lincRNAs, is a complement to the list of other ncRNAs that have already been discovered in this species^30^,^31^, and therefore will help to better annotate the genome of DBM^32^. There is an ever-growing number of reports, which illustrate the role that lncRNAs play in cellular defence mechanisms against a wide range of toxic agents. In this study, we hypothesized that insect lncRNAs may regulate detoxification genes and act as important mediators in the development of insecticide resistance in *P. xylostella.* To support our hypothesis, we produced the expression profile of putative lincRNAs in four insecticide-resistant populations and found that a number of *P. xylostella* lincRNAs were considerably altered in resistant populations. Speculation regarding the biological functions of this class of ncRNAs is increasing and these data will facilitate future studies to unravel the function of lincRNAs in insecticide resistance and other detoxification responses in eukaryotic cells.

## Materials and Methods

### RNA-seq Data preparation

Previously sequenced RNA-seq raw data for *P. xylostella* were downloaded from NCBI Sequences Read Archive with accession number SRA034927 and SRP059463^33^,^34^. Raw data were stripped of adapters using CLC Genomic Workbench version 7.5.1 and reads with quality score of above 0.05 and maximum 2 ambiguous sequences were retained for further analysis.

### Large gap mapping and transcript discovery

The CLC Genomic workbench’s Transcript Discovery plugin was used for long intergenic non-coding RNAs discovery in the *P. xylostella* genome. New transcripts were identified by large gap mapping of 330,410,738 reads of seven RNA-Seq libraries to genomic reference (Px genome assembly version2 available at http://iae.fafu.edu.cn/DBM). We implemented strict mapping criteria (mismatch, insertion and deletion costs: 2: 3: 3 respectively). The minimum similarity and length fraction of 0.9 between a mapped segment and the reference were allowed in mapping criteria. The large gap mapper algorithm also requires that each mapped segment must comprise at least 10% of the read and must be of minimum length 17 bp. We considered a gap with maximum 50 Kbp distance between mapped read segments to span the introns from RNA-seq data. The annotations are generated by examining the read mapping and identifying likely regions of genes, their exons and splice sites. The algorithm scanned each gap in the read mapping to see if the gap is assigned to a valid splice site or can be moved to a valid splice site without cost.

### lincRNA identification pipeline

**Figure 1:**
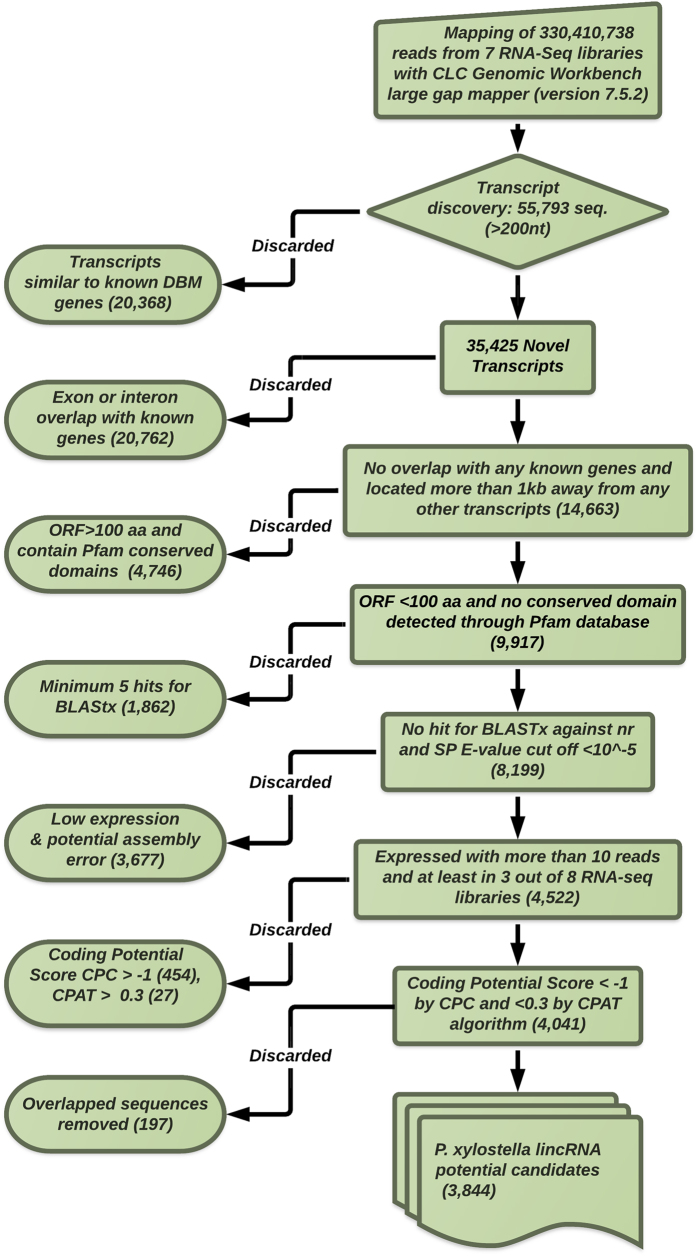
The lincRNA identification pipeline flowchart.

A stringent filtering pipeline was developed to discard transcripts with evidence for protein coding potential. The pipeline for *P. xylostella* lincRNAs discovery is summarized in Fig. 1. We identified 55,793 potential genes using the CLC Genomic Workbench transcript discovery algorithm. The genes that were annotated as known *P. xylostella* genes were discarded and 35,425 potential genes were also checked for any exon or intron overlap with other known *P. xylostella* genes. We selected 14,663 sequences, which were located more than 1 kb away from any other known transcripts, for finding putative open reading frames (ORF). All possible six frames were produced for all selected sequences and then the translated sequences were subjected to a domain search to identify any putative conserved protein domains through Pfam v27.0 database^35^. We discarded 4,746 sequences with potential ORF above 100aa or conserved protein domains. Any possible similarity with other known proteins was found by using BLASTx algorithm against nr and Swiss port database (E-value cut off 10^−5^) for 9,917 of sequences. We also implemented an expression threshold on our data to strengthen the identification pipeline. Sequences with more than 10 mappable reads in at least three out of eight RNA-seq libraries were considered as valid sequences and were kept for the next step. 4,522 sequences were subjected to Coding Potential Calculator (CPC) tool, which is publically available on http://cpc.cbi.pku.edu.cn to check for any other potential coding regions^36^. CPC is a Support Vector Machine-based classifier, which is able to assess the protein-coding potential of a transcript based on six biologically meaningful sequence features. Sequences with the score of above −1 were determined as putative protein coding genes and removed from the list. The data were also submitted to another coding potential assessment tool (CPAT), which uses a logistic regression model built with four sequence features: ORF size, ORF coverage, Fickett TESTCODE statistic and hexamer usage bias^37^. We applied the coding probability threshold of 0.3, which led to discarding 27 sequences as putative coding RNAs. Finally, identical and overlapped sequences were removed from the Px lincRNAs’ profile, and 3844 potential lincRNAs were used for further study.

To identify *P. xylostella* putative lincRNAs that are regarded as small RNA associated lincRNAs, we used the Blast algorithm to search for DBM pre-miRNA sequences in the predicted DBM lincRNA dataset.

### Px lincRNAs expression analysis

The *P. xylostella* genome was annotated with the final list of lincRNAs and used as reference for RNA-seq analysis in CLC Genomic Workbench. To measure the normalized value for RNA expression and remove technical biases, which is natural in the sequencing approach, RPKM (Reads Per Kilobase per Million reads) was assigned as expression value in each library^38^. To find the differential expression pattern in the insecticide resistant population, data from Fipronil (FR) and Chlorpyrifos (CR) resistant larvae were compared with the insecticide-susceptible strain larvae^33^. We also downloaded the *P. xylostella* midgut transcriptome data from *Bacillus thuringiensis* (Bt) resistant populations through SRA074406 and compared the expression values of the putative lincRNAs from two Bt-resistant strains (MK and GK) with those of a Bt-susceptible (MM) population^39^. Baggerley’s test, a count based statistical analysis was done on the data. The samples were given different weights depending on their sizes (total counts). The weights were obtained by assuming a Beta distribution on the proportions in a group, and estimating these, along with the proportion of a binomial distribution.

The nearest neighbouring genes to the set of 3844 putative lincRNAs were extracted using Px genome assembly version 2 coordinates and their expression values were calculated by CLC Genomic Workbench in a similar way. The gene ontology (GO) enrichment was extracted for all the neighbouring protein-coding genes and more abundant terms were computed for each category of molecular function, biological process and cellular components.

### Resistance selection and validation of Px lincRNA expression

To generate an insecticide-resistant *P. xylostella* population, a population of *P. xylostella* collected from a cabbage field in Gatton, south-east Queensland in early 2014 was exposed to deltamethrin (Decis®, 25 g/l EC, Bayer) in the laboratory for 15 generations. Based on comparison of LC_50_ values for this selected population (LC_50_: 1291.59 ppm; 95% CI: 1145.09–1496.93 ppm and slope ± SE: 9.189 ± 1.934) and the Waite insecticide-susceptible population (LC_50_: 0.519 ppm; 95% CI: 0.244–1.413 and slope ± SE: 0.98 ± 0.20), that has been maintained in the laboratory without exposure to any insecticides for more than 25 years^40^, this population demonstrated 2500-fold resistance to deltamethrin.

To determine the relative transcript levels of a selected number of lincRNAs in insecticide-resistant and insecticide-treated *P. xylostella*, thirty 3^rd^ instar larvae from the deltamethrin-resistant population (Delta-R) were exposed to 1000 ppm deltamethrin (~LC_50_ of the previous generation) and the surviving larvae were collected 24 h post-treatment. In the control groups, we included insects from the Delta-R and Waite populations that had not been exposed to insecticide.

Total RNA was extracted from all larval samples using TRIzol® reagent (Life Technologies). Concentrations of the RNA samples were measured using a spectrophotometer and integrity was ensured through analysis on a 1% (w/v) agarose gel. First strand cDNA was synthesized from 2 μg of RNA using a poly-dT primer and Superscript III reverse transcriptase (Life Technologies). The follow up qPCR reaction consisted of 2 μL of diluted cDNA (10 ng), 5 μL of QuantiFast SYBR Green PCR Master Mix with ROX (Qiagen), and 1 μM of each lincRNA specific (forward and reverse) primers (Supplementary Table S1) in 10 μL total volume. Reactions were performed in triplicates in a Rotor-Gene thermal cycler (Qiagen) under the following conditions: 95 °C for 5 min; and 40 cycles of 95 °C for 10 s and 60 °C for 30 s, followed by the melting curve (68 °C to 95 °C). Melting curves for each sample were analysed after each run to check the specificity of amplification. Gene copy numbers were calculated using the Rotor-Gene software and an endogenous reference *actin* gene was used for normalization. One-way ANOVA with Tukey’s PostHoc test was used to identify statistically significant differences.

## Results and Discussion

### Identification and characterisation of *P. xylostella* lincRNAs

**Figure 2:**
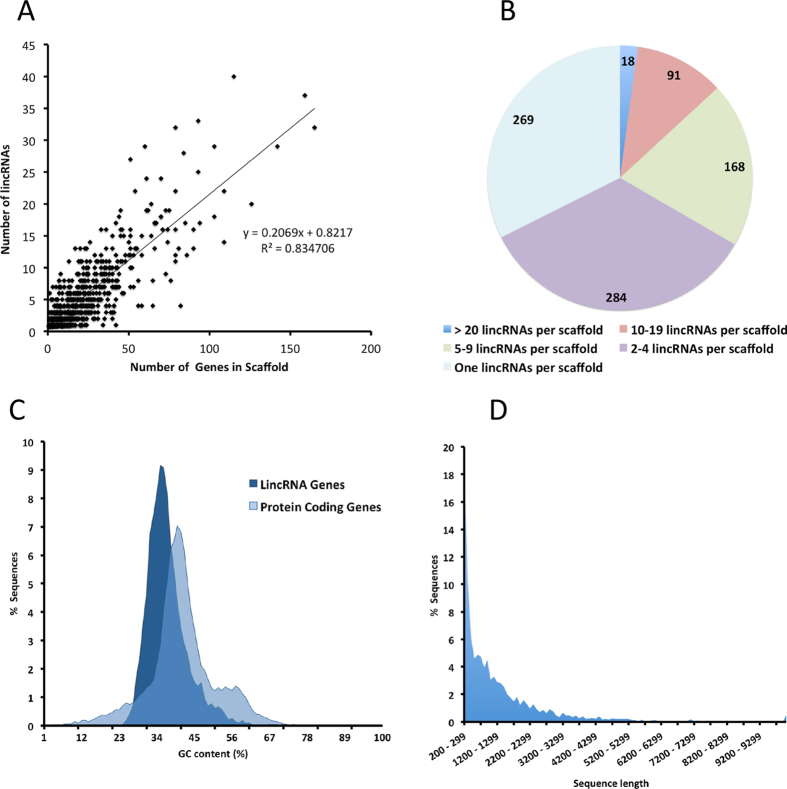
*Plutella xylostella* lincRNA characterization. (**A**) There are significant correlations between the number of protein coding genes and lincRNAs in each genome scaffold. (**B**) The majority of scaffolds only contain 1–4 lincRNAs while only 18 *P. xylostella* genome scaffolds contain more than 20 lincRNAs. (**C**) Comparison of the GC content in protein coding genes and putative lincRNA genes. (**D**) Size distribution of *P. xylostella* lincRNA candidates.

The *P. xylostella* genome is not completely annotated and its chromosome arrangement is not available, however, larger scaffolds with more predicted genes were found to have more lincRNA than shorter scaffolds. A positive correlation (R^2^ = 0.834, *P* < 0.0001) was found between the number of genes on scaffolds and putative lincRNAs (Fig. 2A). In total, 3,844 putative lincRNAs in 830 *P. xylostella* genome scaffolds were identified (Supplementary File 1). The majority of these scaffolds contain only one lincRNA locus (34%), however, 18 scaffolds (2%) were enriched with more than 20 lincRNAs (Fig. 2B). The detailed information of these scaffolds, which contain the highest number of lincRNAs are summarized in Table 1.

The DBM lincRNA genes displayed a slightly lower GC content (average 36.9%) in comparison to 42.0% in their protein coding gene sequences (Fig. 2C). The lower GC content or AT enrichment is a typical characteristic of lincRNAs and our findings are congruent with other predicted lincRNAs in other species^41^,^42^. However, it has been shown that lncRNAs with higher GC content are marginally more stable and they displayed an increased half-life in mouse Neuro-2a cell line^42^. Global measurement of half-lives of lncRNA in mouse neural cells exhibited a universal instability for lncRNAs as compared with mRNAs^42^, however, among different classes of lncRNAs, intergenic transcripts are more stable than intronic lncRNAs^43^.

**Figure 3:**
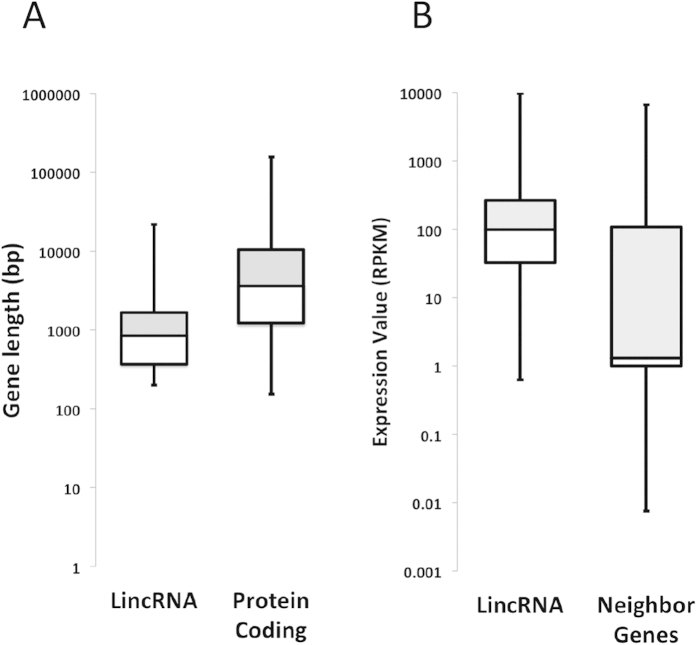
Comparison of gene length and expression value in *P. xylostella* lincRNAs and protein coding genes. (**A**) Gene length comparison, (**B**) The expression value (RPKM) of the neighbouring genes.

The short transcript and gene lengths, low exon number and lower expression, compared to protein-encoding genes are typical characteristics of mammalian lncRNAs^2^,^24^. The majority of *P. xylostella* predicted lincRNAs are smaller than 4000 bp and their length distribution is represented in Fig. 2D. *Plutella xylostella* lincRNA candidates are notably shorter in length than protein-coding genes, demonstrating another well known characteristic of lncRNA transcripts (Fig. 3A). We measured the gene expression level (RPKM) of all the identified lincRNAs and their proximity to protein-coding transcripts. The data showed that overall the neighbouring protein coding genes are expressed much less than the *P. xylostella* predicted lincRNAs (Fig. 3B), this is in contrast to the current literature in mammalian species. However, Cabili *et al.* (2011) found that only 28% of their identified lincRNAs had a significant correlation with their neighbouring protein coding genes^2^, and it has also been shown that they have strong tissue-specific expression patterns. In addition, it has been demonstrated that in *MALAT1* knockout mice, in which one of the most abundant and conserved lncRNAs (*MALAT1*) is absent, only a small number of its neighbouring genes were deregulated^43^,^44^.

**Figure 4:**
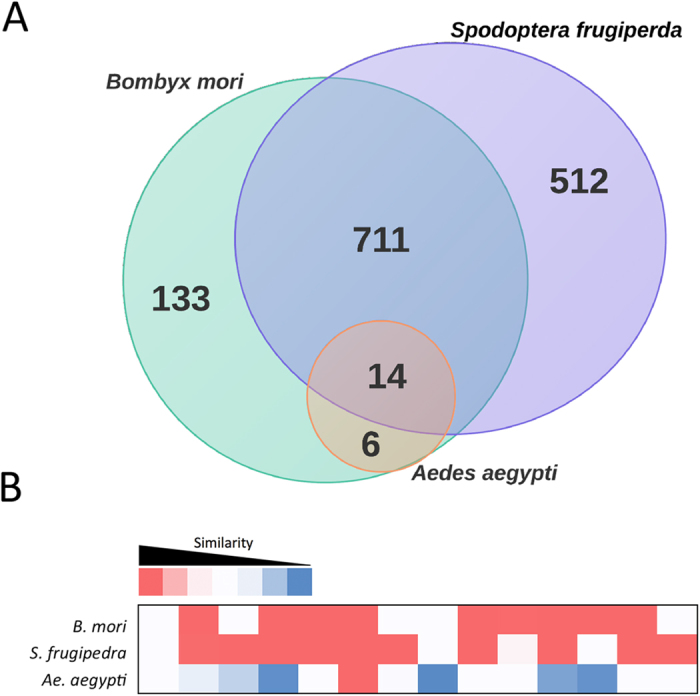
*P. xylostella* lincRNAs share some conserved areas with other closely related species. The Venn diagram displays the number of DBM lincRNAs with similarity above the cut off in other species. (**B**) The heat map of 14 overlapped sequences based on E-value shows more similarity between DBM lincRNA with two closely related species.

We found some level of similarity among *P. xylostella* lincRNA sequences with other closely related insect genomes such as *Bombyx mori*, *S. frugiperda* and one distantly related mosquito, *Aedes aegypti* (Fig. 4). The E-value cut off 10^−10^ was applied to our screening with the BLAST algorithm to identify the conserved sequences among *P. xylostella* lincRNAs and the three other insect genomes. Although the DBM *P. xylostella* lincRNAs shared many sequences with genomes of the two closely related insects (864 and 1243 sequences with conserved areas in *B. mori* and *S. frugiperda* genomes, respectively), only 14 had detectable sequence similarity among all three species and were usually limited to a single short region of high conservation (Fig. 4). Ulitsky *et al.* (2011) identified 550 distinct lincRNAs in zebrafish, but only 29 showed similar sequence conservation with their putative mammalian orthologues^45^. Previous studies reported thousands of lincRNAs in human and other model organisms, while just a few have detectable sequence homology in other species. Lack of conservation among identified lincRNAs is one of the challenges in their comparative sequence analysis and functional studies among non-model organisms^45^.

In a recent annotation of DBM genome in NCBI, 707 loci were predicted as lncRNAs with Gnomon, a NCBI eukaryotic gene prediction tool^46^. In the current study, we recalled 250 of those sequences in the *P. xylostella* lincRNA repertoire with BLAST E-value cutoff of 10^−10^ and minimum bit score of 100, which share some conserved or repetitive regions. Nevertheless, only 78 newly predicted lincRNAs overlapped with the recently annotated *P. xylostella* lncRNAs and were located in the similar locus. Several *P. xylostella* lincRNAs also share repetitive sequences or previously identified microsatellite sequences^47^. It has been shown that many repetitive elements such as telomeric RNAs, satellite RNAs and even some retrotransposons are among lncRNAs. There is significant evidence of similar repetitive non-coding sequences in mammals, whose expressions are altered by external stimuli^48^. For example, transcripts of satellite RNA repeats within the chromatin could act as *cis* regulators^49^. lincRNAs also play a role in the regulation of the neighbouring genes and act as *cis*-regulatory elements, however, they also have the ability to act in *trans* and regulate distantly located genes.

**Figure 5:**
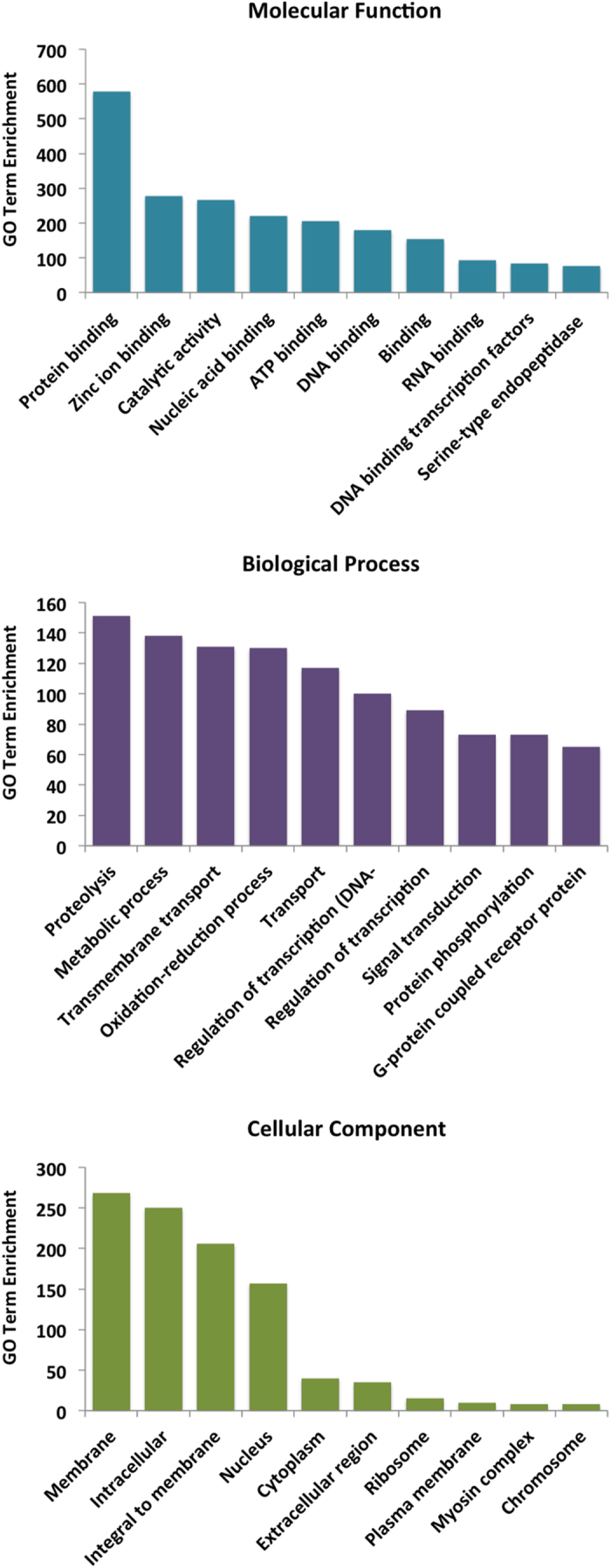
Gene function enrichment analysis based on Gene Ontology (GO) annotation of putative *P. xylostella* lncRNA’s proximal genes.

In general, lincRNAs are co-expressed with their neighbouring genes^2^. Typically they are transcribed from the chromosome that they are regulating^6^. To functionally characterize *P. xylostella* lincRNAs, gene ontology (GO) enrichment of their associated protein-coding genes was extracted. The GO terms enrichment in molecular function category indicate that *P. xylostella* lincRNAs are likely to play an important role in binding associated activity because most of the overrepresented GO terms are linked to protein binding, zinc ion binding and even DNA and RNA binding functions (Fig. 5). Proteolysis and metabolic process were the most abundant GO terms among Biological Process. The GO term of “Regulation of transcription” was also overrepresented among the Biological Process category, which is a common and the most abundant term among other species of lncRNA GO analysis^11^,^25^,^50^,^51^.

Our results suggest that a small number of *P. xylostella* lincRNAs are involved in the production of small RNAs or serve as primary miRNAs. Previous studies also reported that the majority of lincRNAs are processed by small RNA-independent machinery^4^. We identified 10 miRNA precursors in our lincRNA profile (Supplementary Table S2). The pre-miRNA sequences of recently annotated *P. xylostella* miRNAs miR-8497, miR-8517 and miR-8546^31^ were identified in more than 20 potential lincRNAs, which shows that these miRNAs originate from repetitive elements in the genome.

The different proposed scenarios on the interaction of small RNAs, such as miRNAs, with lncRNAs, have been reviewed by Yoon *et al.* 2014^52^. Long non-coding RNAs can be regulated by some miRNAs. It has been revealed that miRNA let-7b contributed to lowering lincRNA-p21 stability in human cervical carcinoma cells and overexpression of this miRNA may facilitate degradation of the lincRNA^53^. In a recent study, miR-CLIP technology identified lincRNA H19 as a target of miR-106a. Surprisingly, the miRNA overexpression through ectopic delivery of miR-106a into cells caused an approximately 6-fold increase in human lincRNA H19^54^. In other cases, lncRNAs can act as miRNA decoys and also compete with them to bind to mRNAs. Those lncRNAs, which are involved in regulation of gene expression, may compete with miRNAs to control their target genes. As we have shown here, some miRNAs may originate from lncRNAs. It has been shown that human lincRNA MD1 generates miR-206 and miR-133b from an intron and an exon, respectively^55^. To date, most studies have been done in human cells, and mainly on miRNA and lncRNA interactions. More studies are required to fully understand the complexity of interaction between small and long ncRNA and their link to various biological conditions.

### DBM lincRNAs differentially expressed in insecticide resistance larvae

**Figure 6:**
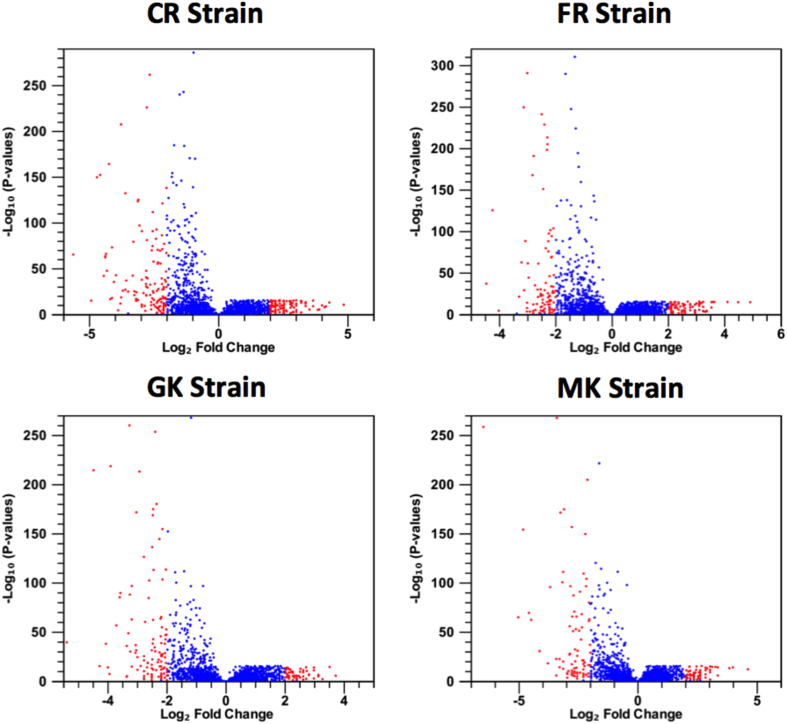
Volcano plot of differentially abundant *P. xylostella* lincRNAs in insecticide-resistant larvae compared with their corresponding control. CR: Chlorpyrifs resistance; FR: Fipronil resistance; MK and GK: two resistance strains to *Bacillus thuringiensis* endotoxins. The red dots represent statistically significant altered lincRNA with more than 4 fold (*P* < 0.05). The blue dots represent lincRNA with less than 4 fold changes in two conductions.

**Figure 7:**
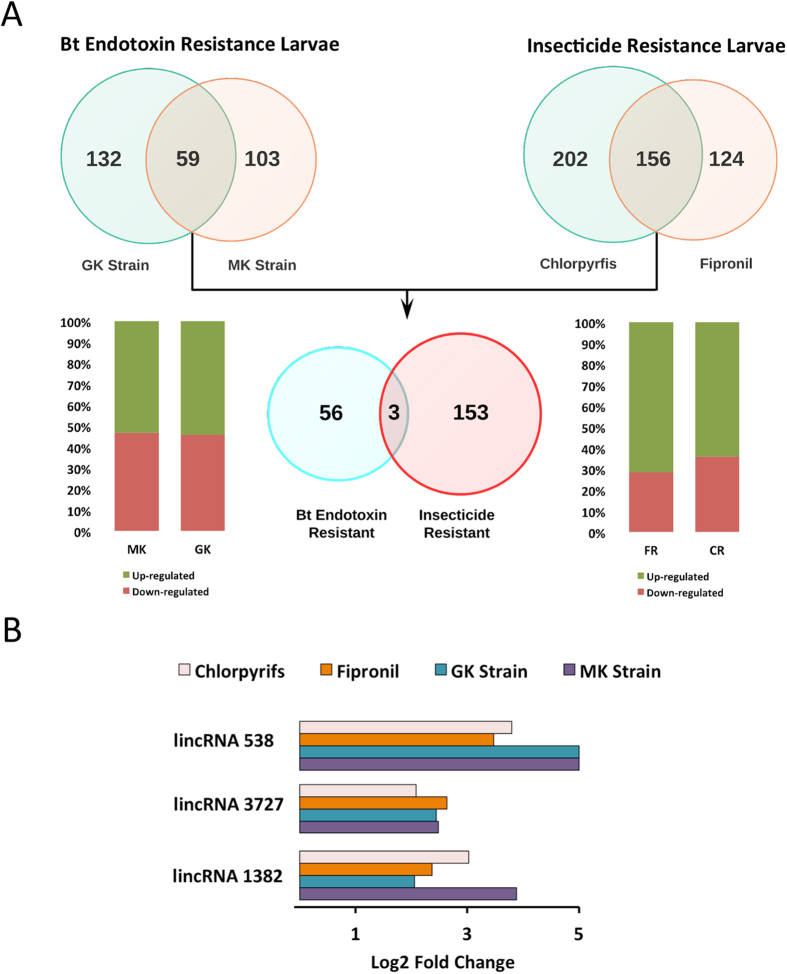
The number of unique and overlapped lincRNAs among different insecticide resistance groups. There were 59 common differentially expressed lincRNAs between two Bt resistance strains and 156 lincRNAs between Chlorpyrifs and Fipronil resistance larvae (**A**). The bar charts show the proportion of up or down regulated genes among each group. There were only three differentially abundant lincRNAs between Bt resistance and other insecticide resistance strains with more than 4-fold up-regulation (**B**).

The RNA-seq data of *P. xylostella* insecticide-resistant populations were available on public databases^33^,^39^. We reanalyzed those data to determine the expression profiles of *P. xylostella* lincRNAs in insecticide-resistant larvae and compare them with the corresponding control groups to explore their differential expression patterns (Supplementary Files 2 and 3). The expression profiles of *P. xylostella* lincRNAs in insecticide-resistant larvae to two classes of insecticides, Fipronil (FR, from the phenylpyrazole family) and Chlorpyrfos (CR, an organophosphate), showed noticeable alteration when compared with those of susceptible larvae (Fig. 6). We also reanalyzed the *P. xylostella* larval midgut transcriptome data in two resistant strains (MK and GK) to *Bacillus thuringiensis* (Bt) endotoxin Cry1Ac to explore alterations in the expression profiles of *P. xylostella* lincRNAs in comparison with those of susceptible larvae (MM)^39^ (Fig. 6). Using strict criteria (Fold change above 4, *P*-value < 0.05 and presence of at least 10 reads in resistance or susceptible strains’ libraries), we found 358, 280, 169 and 191 lincRNA genes differentially expressed (DE) in CR, FR, GK and MK groups, respectively (Fig. 7A). The majority of DE lincRNAs were overexpressed (~70%) in insecticide-resistant populations; however, in Bt resistant strains almost 50% of DE lincRNAs were up-regulated. There were only three lincRNAs (lincRNAs 538, 3727 and 1382), which were noticeably altered in all the four resistant strains (Fig. 7B). We found that distinct sets of lincRNAs were commonly altered between the two insecticide and Bt endotoxin resistant strains, which suggests that this class of RNAs may act differently in cellular defense mechanisms against individual xenobiotic agents. In a previous study, it was shown that two genotoxic drugs, mitomycin C and doxorubicin, altered lncRNA expression profiles in HeLa cells but there were no commonly altered lncRNAs between these two genotoxic components due to their different mode of action^18^.

There is a growing body of evidence indicating that ncRNAs play significant roles in toxicogenomics^56^,^57^,^58^,^59^,^60^. However, there are still only few studies about the impact of xenobiotics on the expression of lncRNAs^61^. The gene expression profile of three well-characterized *Chironomus riparius* lncRNAs, telomeric repeats, Cla repetitive elements and the SINE CTRT1, were examined in response to various types of aquatic contaminants^61^. Transcription levels of telomeric repeats and Cla were increased after bisphenol A (BPA) and heavy metal cadmium (Cd) treatments^61^. A recent study in a mammalian cell line also introduced lncRNA as a new class of targets in neurotoxicity^62^. Their role in a few toxic-mediated neurological diseases has been studied but little is known regarding the potential function that lncRNAs may play in detoxification or other toxic related metabolisms.

### Experimental validation of Px lincRNAs expression

**Figure 8:**
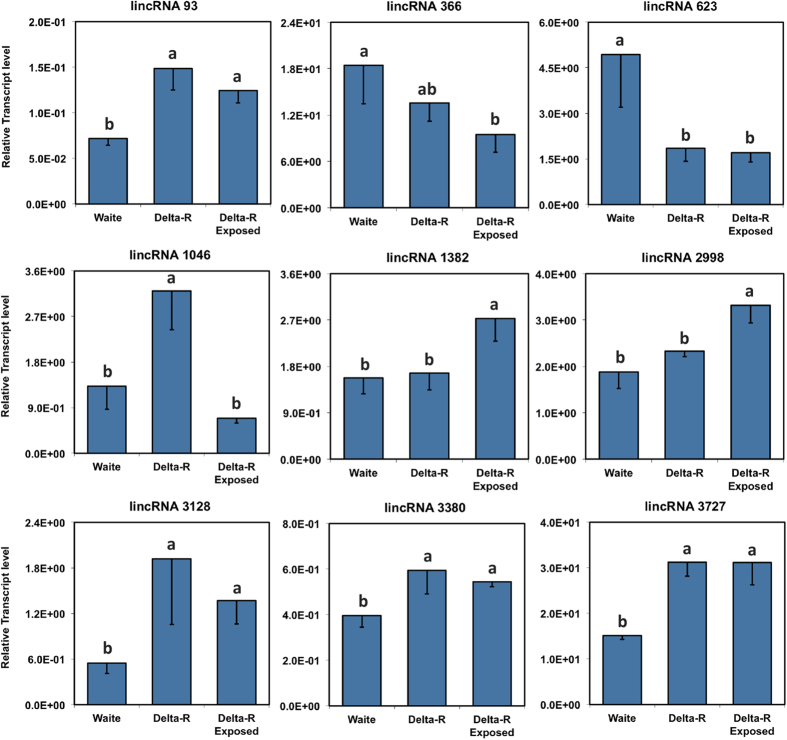
The relative transcript levels of a selected number of DBM lincRNAs in insecticide-resistant (Delta-R) and -susceptible DBM larvae. The expressions levels of lincRNAs were measured using qRT-PCR in RNA extracted from the insecticide-susceptible Waite population (Waite) and Delta-R strains exposed to 1000 ppm deltamethrin to see the direct response of lincRNAs to insecticide treatment. Different letters indicate statistically significant differences at *P* < 0.05.

To validate putative *P*. *xylostella* lincRNAs and their differential expression in insecticide-resistant and insecticide–susceptible larvae, we used qRT-PCR to measure the relative transcript levels of a number of randomly selected lincRNAs (Fig. 8). The majority of putative lincRNAs (lincRNA 93, 1046, 3128, 3380, 3727) showed significant overexpression in deltamethrin-resistant (Delta-R) larvae in comparison with the insecticide susceptible (Waite) larvae. All these up-regulated lincRNAs, except lincRNA 3128, showed considerable overexpression in other insecticide resistant populations based on transcriptome analyses (Supplementary Files 2 and 3). The overexpression of lincRNA 3727 was not only observed in all insecticide-resistant populations (*ie.* those resistant to deltamethrin, fipronil and chlorpyrifos), but also considerable enhancement was detected in both populations resistant to Bt endotoxins (Fig. 7B). Deep sequencing data analysis showed that lincRNA 623 was dramatically down-regulated in resistant larvae and we also found significant suppression of the lincRNA in Delta-R larvae (Fig. 8).

Response to insecticide exposure was also detected in some lincRNAs. When the deltamethrin-resistant (Delta-R) population was examined the transcript levels of lincRNA 1046 drastically dropped in deltamethrin treated larvae in comparison with larvae that were not exposed to the insecticide. Also significant overexpression response to direct treatment was observed in lincRNA 2998 (Fig. 8). Although the roles of the identified lincRNAs remain unknown, these genes have the potential to be involved in insecticide resistance development or detoxification pathways. Insecticide exposure may produce heritable modifications in gene expression that occur without a change in the DNA sequence, which are defined as epigenetic effects. Several epigenetic mechanisms, such as DNA methylation and histone modifications, can be caused by pesticides through lncRNA-mediated pathways^63^,^64^. A few studies have demonstrated the association of long and intermediate ncRNAs in epigenetic modification in distinct insect species^65^,^66^,^67^. For instance in *B. mori*, some intermediate ncRNAs are involved in the repression of transcription in silk gland by epigenetic modifications of histones^67^. Epigenetic effects of dichlorvos, fipronil and triazophos, members of different classes of insecticides, on miRNA profiles and mRNA gene expression of mammalian cells and other model animals such as zebrafish have also been studied^56^,^68^. Based on our knowledge of the current literature, our work is the first study to comprehensively investigate the larval lincRNA profiles in various types of insecticide resistant strains of *P. xylostella*.

## Conclusions

In the current study, we provide a comprehensive list of lincRNAs from *P. xylostella* and show that their expression profile is changed in larvae resistant to three different classes of insecticides, an organophosphate, phenylpyrazole and Bt endotoxins, which have widely different modes of action (inactivate acetylcholinesterase, target GABA-gated chloride channel and lyse midgut cells, respectively). In addition, we observed significant alteration in expression of lincRNAs in insecticide-treated *P. xylostella* larvae, which we speculate may have direct or indirect links to detoxification pathways or stress responses. The general knowledge of the biological functions of lincRNAs in insecticide resistance and detoxification pathways is still limited, but when taken together these results provide further evidence to support the hypothesis that lincRNAs may play a role in insecticide resistance development. We believe, it is important to analyze the lincRNA’s responses in different species to a wider range of insecticides or other xenobiotics, which may help to explain some unknown role(s) of this class of ncRNAs in detoxification or other toxin related metabolisms.

## Additional Information

**How to cite this article**: Etebari, K. *et al.* Genome wide discovery of long intergenic non-coding RNAs in Diamondback moth (*Plutella xylostella*) and their expression in insecticide resistant strains. *Sci. Rep.* **5**, 14642; doi: 10.1038/srep14642 (2015).

## Supplementary Material

Supplementary Information

Supplementary Dataset 1

Supplementary Dataset 2

Supplementary Dataset 3

## Figures and Tables

**Table 1:** Distribution of potential *P. xyllostella* lincRNAs in different scaffolds with more than 20 lincRNAs and their comparison with number of protein coding genes.

Scaffold ID	Length(bp)	No. knowngenes	No.lincRNA	Length range(bp)	Average size oflincRNA (bp)
Scaffold_51	2546611	115	40	202–4513	1268
Scaffold_14	3424285	159	37	222–4001	1153
Scaffold_7	2281484	93	33	221–4544	1099
Scaffold_16	3493687	165	32	206–5659	1028
Scaffold_109	2311385	79	32	214–4763	1314
Scaffold_15	2764502	142	29	201–4806	1188
Scaffold_10	2295158	103	29	206–4171	892
Scaffold_11	1785983	60	29	208–5642	1512
Scaffold_31	2511109	84	28	246–2619	862
Scaffold_5	1890696	51	27	212–5103	1332
Scaffold_21	2450764	93	25	276–4359	1090
Scaffold_12	1974453	61	24	200–4714	1183
Scaffold_52	1725502	70	24	204–5203	1361
Scaffold_8	2584131	109	22	210–7301	2595
Scaffold_4	2074850	79	22	205–3210	928
Scaffold_199	1392491	54	22	210–9894	1311
Scaffold_30	2356637	126	20	220–5718	1192
Scaffold_102	1646965	64	20	203–4765	1259
