# Supplementary material for: Genome wide discovery of long intergenic non-coding RNAs in Diamondback moth (Plutella xylostella) and their expression in insecticide resistant strains
Source: Sci Rep. 2015 Sep 28;5:14642. doi: 10.1038/srep14642 (PMC4585956; doi:10.1038/srep14642)
Supplement: Supplementary Information [file srep14642-s1.pdf]

Title: Genome wide discovery of long intergenic non-coding RNAs in Diamondback moth (*Plutella xylostella*) and their expression profile in insecticide resistant strains

Kayvan Etebari, Michael J. Furlong and Sassan Asgari

Table S1. The primer sequences used for qRT-PCR validation.

Gene ID	Forward primer	Reverse primer
lincRNA_3380	TGGCAACCAGAACAAGAGTG	TACCTGCATCGCTGCATAGAC
lincRNA_3727	AATAGGGAATCGAGGGTTGG	CGAATGATTCAGCACCTAGC
lincRNA_3128	GTGGGCCAAAAGTAATCACC	GGCCTCTGAACTTGTATTCTGC
lincRNA_93	AGCAATAAGGGGGTCAAGTC	TCAGGCAACGATAACCTCAG
lincRNA_623	GTGCCATCTGTTGATCTTCG	TCGGCTGCACTGAAGACTATC
lincRNA_1046	AACCACTAGCCCGTTCATTG	CAACGACTTCGCTGAATGAC
lincRNA_2998	GTGTGTTCCGTGCAAAACTG	CCATGCCAAAGCTAAAGTTG
lincRNA_1382	ACCAACCTTGGCAGTAAACG	TTGCTAGCACGGTCATCATC
lincRNA_366	AGTTCGTGATTGGTCCTTGC	TTTTTCGTTGCCTGCCTAAC
Actin	ATGGAGAAGATCTTGCAC	GGAGCCTCCGTGAGCAGC

Table S2. The miRNA associated lincRNAs.

microRNA	Associated lincRNAs	Pre-miRNA Length (bp)	Greatest hit Length (bp)	Lowest E-value
mir-274	lincRNA_3134	94	94	1.89E-44
mir-8497	lincRNA_2175, lincRNA_3250, lincRNA_1774, lincRNA_3716, lincRNA_1252, lincRNA_0904, lincRNA_3009, lincRNA_1715, lincRNA_0543, lincRNA_1675, lincRNA_1740, lincRNA_0936, lincRNA_3026, lincRNA_3237, lincRNA_3210, lincRNA_1469, lincRNA_2395, lincRNA_2993, lincRNA_0926, lincRNA_3334, lincRNA_0055, lincRNA_0078, lincRNA_2222, lincRNA_2420, lincRNA_3292, lincRNA_1803, lincRNA_1939, lincRNA_2037, lincRNA_3464, lincRNA_0068, lincRNA_0241, lincRNA_1480, lincRNA_3312, lincRNA_0470, lincRNA_2967, lincRNA_3377, lincRNA_3400, lincRNA_0229, lincRNA_0962, lincRNA_1571	188	153	1.03E-50
mir-8499a	lincRNA_0249	77	88	9.64E-36
mir-8500	lincRNA_3208	168	134	4.29E-12
mir-8517a	lincRNA_0125, lincRNA_3295, lincRNA_1274, lincRNA_0027, lincRNA_0046, lincRNA_0083, lincRNA_3833, lincRNA_2414, lincRNA_1941, lincRNA_0769, lincRNA_3053, lincRNA_3190, lincRNA_1305, lincRNA_2869, lincRNA_2098, lincRNA_2351, lincRNA_1182, lincRNA_0994, lincRNA_2169, lincRNA_0709, lincRNA_0587, lincRNA_1287, lincRNA_2360, lincRNA_2529, lincRNA_3783, lincRNA_0457, lincRNA_1879, lincRNA_0302, lincRNA_0206, lincRNA_1077, lincRNA_1048	173	168	4.65E-55
mir-8517b	lincRNA_2769, lincRNA_0880, lincRNA_2843, lincRNA_3603	142	164	2.52E-54
mir-8546	lincRNA_0427, lincRNA_0278, lincRNA_0408, lincRNA_0646, lincRNA_0698, lincRNA_0700, lincRNA_0833, lincRNA_0941, lincRNA_1046, lincRNA_1282, lincRNA_1586, lincRNA_1707, lincRNA_1762, lincRNA_1779, lincRNA_1923, lincRNA_2223, lincRNA_2669, lincRNA_2808, lincRNA_2816, lincRNA_3363, lincRNA_3733, lincRNA_3749, lincRNA_3819, lincRNA_3055	76	70	2.69E-30
mir-965	lincRNA_0214	94	94	2.82E-44
mir-9a	lincRNA_1147	94	94	1.20E-44
pxy-let-7	lincRNA_0250	115	115	4.15E-56
